# ROTAS: a rotamer-dependent, atomic statistical potential for assessment and prediction of protein structures

**DOI:** 10.1186/1471-2105-15-307

**Published:** 2014-09-18

**Authors:** Jungkap Park, Kazuhiro Saitou

**Affiliations:** Department of Mechanical Engineering, University of Michigan, Ann Arbor, MI USA

## Abstract

**Background:**

Multibody potentials accounting for cooperative effects of molecular interactions have shown better accuracy than typical pairwise potentials. The main challenge in the development of such potentials is to find relevant structural features that characterize the tightly folded proteins. Also, the side-chains of residues adopt several specific, staggered conformations, known as rotamers within protein structures. Different molecular conformations result in different dipole moments and induce charge reorientations. However, until now modeling of the rotameric state of residues had not been incorporated into the development of multibody potentials for modeling non-bonded interactions in protein structures.

**Results:**

In this study, we develop a new multibody statistical potential which can account for the influence of rotameric states on the specificity of atomic interactions. In this potential, named “rotamer-dependent atomic statistical potential” (ROTAS), the interaction between two atoms is specified by not only the distance and relative orientation but also by two state parameters concerning the rotameric state of the residues to which the interacting atoms belong. It was clearly found that the rotameric state is correlated to the specificity of atomic interactions. Such rotamer-dependencies are not limited to specific type or certain range of interactions. The performance of ROTAS was tested using 13 sets of decoys and was compared to those of existing atomic-level statistical potentials which incorporate orientation-dependent energy terms. The results show that ROTAS performs better than other competing potentials not only in native structure recognition, but also in best model selection and correlation coefficients between energy and model quality.

**Conclusions:**

A new multibody statistical potential, ROTAS accounting for the influence of rotameric states on the specificity of atomic interactions was developed and tested on decoy sets. The results show that ROTAS has improved ability to recognize native structure from decoy models compared to other potentials. The effectiveness of ROTAS may provide insightful information for the development of many applications which require accurate side-chain modeling such as protein design, mutation analysis, and docking simulation.

**Electronic supplementary material:**

The online version of this article (doi:10.1186/1471-2105-15-307) contains supplementary material, which is available to authorized users.

## Background

Understanding the structure and function of proteins requires an accurate potential energy function to quantify interactions between residues or atoms. One approach for the design and construction of potential energy functions is to make use of the information embedded in the known protein structures [1–6]. Such energy functions, called statistical potentials or knowledge-based potentials are derived by converting the observed frequencies of residue or atomic interactions in a database of protein structures into the free energies of corresponding interactions. Any aspect of structural features which characterize important interactions in the folded structures can be incorporated into the derivation of statistical potentials. Although their physical interpretations are still debated [7–9], due to their accuracy and computational efficiency, statistical potentials have been used with considerable success in many applications such as fold recognition and threading [10, 11], protein structure prediction [12], protein design [13], binding [14, 15] and aggregation [16].

The key idea in the development of statistical potentials is how to decompose the 3-D network of interactions in protein structures. Typical pairwise potentials cannot accurately describe non-bonded interactions in protein structures. As the folded protein structures are tightly packed and surrounded by solvent molecules, the surrounding circumstances of interacting atoms are inhomogeneous and anisotropic. Also, due to the bond connectivity, there are always correlated interactions from nearby bonded atoms. Thus, more detailed and complex structural features involving multibody effects have been incorporated into the formulation of statistical potentials. For example, sequential segments of various lengths have proved useful for prediction of secondary structure [17–20]. Four body potentials were used to improve cooperativity of main-chain hydrogen-bonds [21, 22]. A variety of structural motifs (i.e., residue clusters) has been identified to better characterize tightly packed protein structures [23–28]. Delaunay tessellation technique also has been employed as a means of defining multibody interactions [29, 30]. Local environment templates which could account for maximum 17 residues have been introduced to more accurately capture cooperative effects in protein structures [27]. A secondary structure specific implementation of pairwise potentials has demonstrated its superiority to typical residue pairwise potentials [31, 32]. The introduction of orientation dependencies of interactions into typical distance-dependent pairwise potentials has achieved substantial improvements in both residue-level [33–36] and atomic-level potentials [37–40]. These multibody potentials are not only able to describe the 3-D interactions more completely but also able to account for cooperative effects of molecular interactions more accurately than typical pairwise potentials.

On the other hand, protein residues have great flexibility because their single covalent bonds allow rotation of the atoms they join. It is well known that residues prefer to adopt only a limited number of staggered conformations, known as rotamers due to local steric interactions (e.g. overlapped electron orbitals) [41–44]. Since the electron density distribution around each nucleus can vary depending on the molecular conformation [45–47], different rotamers may result in different dipole moments and induce charge reorientations, which are reflected in dispersion forces and electrostatic forces. In addition to the polarization effect, solvation effect may be another source of multibody effects related to the rotameric state. For example, compact rotameric states would prefer to be buried within protein structures, while extended rotameric states would prefer to be exposed to solvent with high conformational entropy. Thus, non-bonded interactions between residue atoms may be influenced by the rotameric state of the residues to which the interacting atoms belong.

Existing potentials had not modeled the flexibility of residues explicitly. For example, residue-level potentials which have only one interaction site per residue simply ignore the flexibility of residue conformation. In case of atomic-level potentials, although the orientation dependence of atomic interactions may be able to account for the anisotropic environment around each atom, it is also based on rigid blocks [37] or rigid atom fragments (i.e. three atoms that are consecutively bonded) [38–40]. Thus they cannot reflect the influence of rotameric states on the specificity of atomic interactions no matter how complete a description of the relative orientation and distance between interacting atoms may be used.

Here we studied the energy dependence of residue flexibility and developed a new multibody potential, named “rotamer-dependent atomic statistical potential” (ROTAS). The interaction between two atoms is specified by not only the distance and relative orientation but also by two state parameters which concern the rotameric state of the residues to which the interacting atoms belong. It was clearly found that the rotameric state of residues is correlated to the specificity of interactions within protein structures. Furthermore, such rotamer-dependencies are not limited to specific type or certain range of interactions. We tested ROTAS on various sets of decoys and compared its performance to those of several existing atomic potentials. The results show that ROTAS led to an improvement not only in the native structure recognition, but also in the best model selection and the correlation coefficients between energy and model quality. The ROTAS potential is freely available in https://sites.google.com/a/umich.edu/rotas/.

## Methods

### Derivation of ROTAS

**Figure 1:**
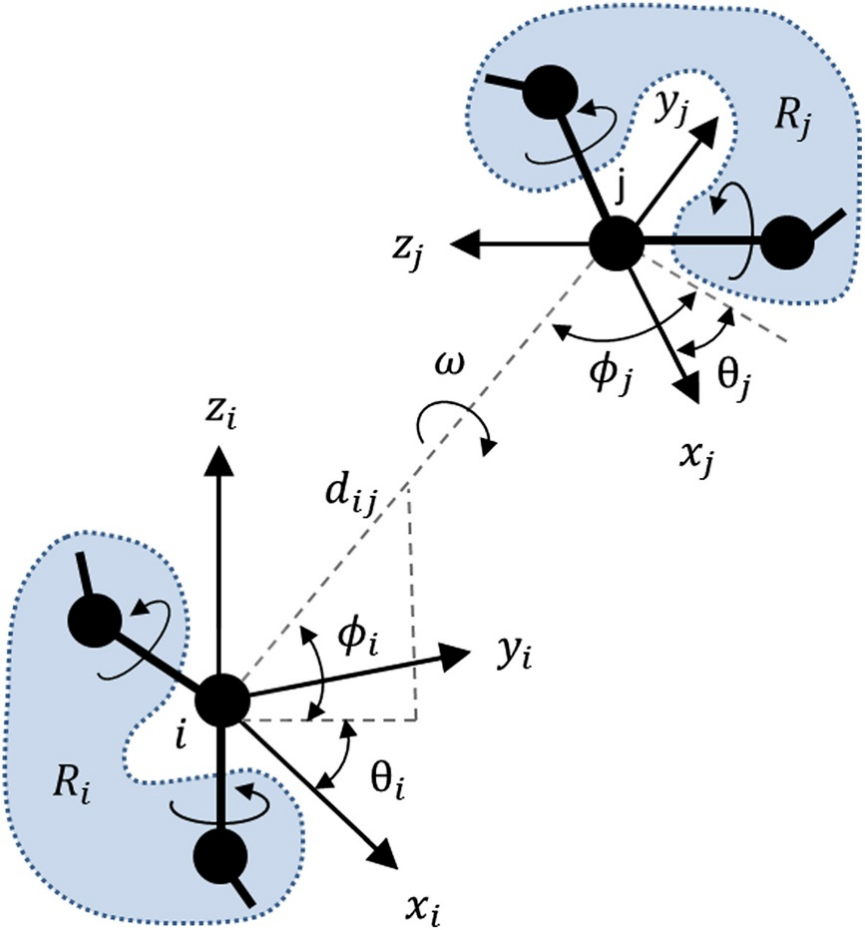
**Description of the interaction between atom types** ***i*** **and** ***j*** **.** Total eight parameters are used to specify the interaction between two atoms. Here, *d*_*ij*_, *θ*_*i*_, *ϕ*_*i*_ are the spherical coordinates of atom *j* with respect to the local frame of atom *i*, and ω is a torsional angle around *d*_*ij*_, and *R*_*i*_ and *R*_*j*_ represent the rotameric state of residues. The rotameric states are determined by side-chain dihedral angles.

In the ROTAS potential, the interaction between two atoms is described by the spatial distance, relative orientation and rotameric states as illustrated in Figure 1. Basically, it extends the description of inter-atomic interaction in GOAP [40] by including the rotameric states of residues. The detailed description for how the rotameric state is defined is explained in the next section. Here we focus on the formulation of the ROTAS potential.

**Figure 2:**
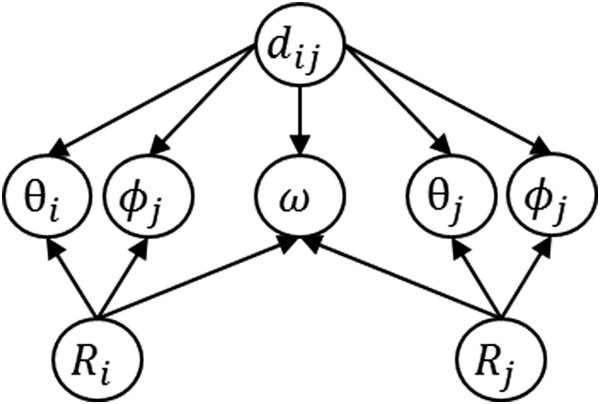
**Bayesian network structure representing conditional independence of variables defined in the ROTAS potential.** The angular parameters are assumed as independent of each other at the given distance and rotameric states.

In this study, we only consider the interaction between heavy atoms and distinguish 167 residue-specific heavy atom types. First, local coordinate frames are attached to all heavy atoms as described in the GOAP potential. The interaction between atom *i* and *j* is then specified by eight parameters: *d*_*ij*_, *θ*_*i*_, *ϕ*_*i*_, *θ*_*j*_, *ϕ*_*j*_, *ω*, *R*_*i*_ and *R*_*j*_ (see Figure 1). Here, *d*_*ij*_, *θ*_*i*_, *ϕ*_*i*_ are the spherical coordinates of atom *j* with respect to the local frame of atom *i*, and ω is a torsional angle around *d*_*ij*_, and *R*_*i*_ and *R*_*j*_ represent the rotameric state of residues. The equation of the ROTAS potential can be obtained using the inverse Boltzmann law:
1

where *k*_*B*_ is the Boltzmann constant and T is the absolute temperature. *P*^*obs*^ is the probability of a particular state (*d*_*ij*_, *θ*_*i*_, *φ*_*i*_, *θ*_*j*_, *φ*_*j*_, *ω*, *R*_*i*_, *R*_*j*_) observed in a sample of known protein structures and *P*^*exp*^ is the expected probability of the same state in a reference state where the interaction is zero. Considering that there are a finite number of known protein structures, we assume conditional dependencies of parameters as shown in Figure 2 to obtain sufficient statistics. Namely, the angular parameters are assumed as independent of each other at the given distance and rotameric states like other studies [38, 40, 48]. Thus the joint probability can be written as
2

Applying Bayes’ rule, the conditional probability, *P*(*ω*|*d*_*ij*_, *R*_*i*_, *R*_*j*_) can be rewritten as
3

Integrating Equation (1)-(3) gives the final equation for the ROTAS potential energy function:
4

Here, *E*(*R*_*i*_) and *E*(*R*_*j*_) can be seen as rotamer intrinsic energy. Assuming that the stability of overall folded structure is mainly determined by non-bonded interactions, we ignore these terms in this study.

### Defining the rotameric state

**Figure 3:**
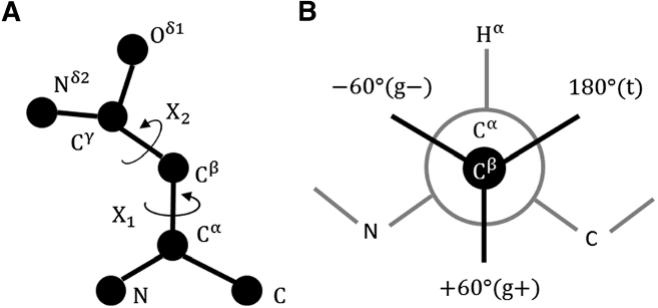
**Schematic representation of residue flexibility and rotameric state. (A)** Ball-stick representation of Asn which has two side-chain dihedral angles. **(B)** Newman diagram of three favored X_1_ angles in proteins. The −60, +60, and 180 angles are often referred to as gauche minus (g-), gauche plus (g+), and trans (t), respectively.

A rotameric state is a combination of side-chain dihedral angles that describes the residue conformation, assuming the bond lengths and angles are fixed (see Figure 3). The observed side-chain dihedral angles cluster around ideal values, such as +60°, −60°, and 180° dihedral angles expected between two sp3 hybridized atoms (see Figure 3B). Since long residues such as Met, Lys or Arg have too many rotameric states to obtain sufficient statistics for each rotamer, we associate up to two side-chain dihedral angles whose rotating bonds are within 3 bond lengths from the considered atom to its rotameric state. For example, the local structural environment of C^β^, C^γ^ and C^δ^ atoms in Lys is defined by a combination of {X_1_, X_2_}, {X_2_, X_3_} and {X_3_, X_4_} dihedral angles, respectively. One exception is the backbone oxygen atom, which is related to {X_1_, X_2_} angles because it frequently interacts with side-chain atoms depending on backbone ψ angle. Also, every atom in Pro is associated to only X_1_ angle because X_2_ angle is strongly correlated with X_1_ angle.

For each side-chain dihedral angle, we divide the dihedral angle space into three or two regions. The dihedral angle between two sp3 hybridized atoms is classified into three distinct rotameric states: 0° ~ 120° (g+), −120° ~ 0° (g-), and 120° ~ 240° (t). Last dihedral angles of Asn, Asp, Gln, Glu, His, Trp, Phe and Tyr are non-rotameric [49]. For those non-rotameric dihedral angles, we divide the dihedral angle space into two regions, {(0 ~ π), (−π ~ 0)}. X_1_ dihedral angle of Pro is also divided into two regions, positive or negative. All 167 heavy atom types and their associated dihedral angles for defining the local structural environments are listed in Table 1.

**Table 1:** **All 167 residue-specific heavy atom types and associated side-chain dihedral angles for defining their rotameric states**

Amino acids	Dihedrals	Associated atoms	Number of rotameric states
GLY	-	C, O, N, C^α^	1
ALA	-	C, O, N, C^α^, C^β^	1
CYS	χ_1_	C, O, N, C^α^, C^β^, S^γ^	3
SER	χ_1_	C, O, N, C^α^, C^β^, O^γ^	3
THR	χ_1_	C, O, N, C^α^, C^β^, O^γ1^, O^γ2^	3
PRO	χ_1_	C, O, N, C^α^, C^β^, C^γ^, C^δ^	3
VAL	χ_1_	C, O, N, C^α^, C^β^, C^γ1^, C^γ2^	3
ILE	χ_1_, X_2_	C, O, N, C^α^, C^β^, C^γ1^, C^γ2^, C^δ1^	9
LEU	χ_1_, X_2_	C, O, N, C^α^, C^β^, C^γ^, C^δ1^, C^δ2^	9
ASP	χ_1_, X_2_	C, O, N, C^α^, C^β^, C^γ^, O^δ1^, O^δ2^	6
ASN	χ_1_, X_2_	C, O, N, C^α^, C^β^, C^γ^, O^δ1^, N^δ2^	6
GLU	χ_1_, X_2_	C, O, N, C^α^, C^β^, C^γ^	9
	χ_2_, X_3_	C^δ^, O^ϵ1^, O^ϵ2^	6
GLN	χ_1_, X_2_	C, O, N, C^α^, C^β^	9
	χ_2_, X_3_	C^γ^, C^δ^, O^ϵ1^, N^ϵ2^	6
MET	χ_1_, X_2_	C, O, N, C^α^, C^β^	9
	χ_2_, X_3_	C^γ^, S^δ^, C^ϵ^	9
ARG	χ_1_, X_2_	C, O, N, C^α^, C^β^	9
	χ_2_, X_3_	C^γ^	9
	χ_3_, X_4_	C^δ^, N^ϵ^, C^ξ^	9
	χ_4_	H^η1^, H^η2^	3
LYS	χ_1_, X_2_	C, O, N, C^α^, C^β^	9
	χ_2_, X_3_	C^γ^	9
	χ_3_, X_4_	C^δ^, C^ϵ^, N^ξ^	9
HIS	χ_1_, X_2_	C, O, N, C^α^, C^β^, C^γ^, N^δ1^, C^δ2^	6
	χ_2_	C^ϵ1^, N^ϵ2^	2
PHE	χ_1_	C, O, N, C^α^, C^β^	3
	χ_1_, X_2_	C^γ^, C^δ1^, C^δ2^	6
	X_2_	C^ϵ1^, C^ϵ2^, C^ξ^	2
TRP	χ_1_, X_2_	C, O, N, C^α^, C^β^, C^γ^, C^δ1^, C^δ2^	6
	χ_2_	N^ϵ1^, C^ϵ2^, C^ϵ3^, C^ξ2^, C^ξ3^, C^η2^	2
TYR	χ_1_	C, O, N, C^α^, C^β^	3
	χ_1_, X_2_	C^γ^, C^δ1^, C^δ2^	6
	χ_2_	C^ϵ1^, C^ϵ2^, C^ξ^, O^η^	2

### Construction of distance-dependent potential

In ROTAS, the distance-dependent pairwise energy term does not involve the rotamer-dependence. While the observed distance-dependent pairwise probability *P*^*obs*^(*d*_*ij*_) can be calculated straightforwardly, a reference state needs to be defined to compute the expected probability *P*^*exp*^(*d*_*ij*_). Because the focus of this work is the effect of rotamer-dependence on the performance of potential energy function, we simply employed the DFIRE [50] reference state. The DFIRE reference state is an ideal gas system in which atoms are uniformly distributed, and has been successfully applied in other studies [38, 40]. The DFIRE-based distance-dependent potential energy can be calculated by
5

where *N*^*obs*^(*d*_*ij*_) is the number of observed atom pair *i* and *j* at distance *d*, and α is a scaling factor such that *N*^*exp*^(*d*) increases in *d*^α^. Beyond a distance cutoff , it is assumed that both observed and expected pairwise distributions are equal. Here we set  = 15 Å and α = 1.61 as suggested by the original work [50]. To obtain the distribution, the bin width is set to 0.5 Å from 0 to 15 Å. When estimating the observed probability and evaluating the distance-dependent pairwise potential, atom pairs that are in the same residue are excluded.

In addition to DFIRE, we constructed other widely used distance-dependent potentials such as RAPDF [51], KBP [52], DOPE [53] and RW [39] and tested each of them in ROTAS in order to examine the influence of different reference states on the performance of ROTAS. The same structural database, distance cutoff and bin width were applied.

### Construction of orientation-dependent potential

In order to obtain smooth and continuous estimates of the observed probability distribution of angular parameters {*θ*_*i*_, *φ*_*i*_, *ω*} for a particular distance and rotameric state (*d*_*ij*_, *R*_*i*_) from a finite sample data, we employed kernel density estimation. Suppose that {*θ*_*s*_}_*s* = 1 … *N*_ is a set of angles θ*i* collected at a given distance *d*_*ij*_ and rotameric state *R*_*i*_. Then the probability density *p*(*θ*_*i*_|*d*_*ij*_, *R*_*i*_) can be calculated using von Mises distribution as the kernel:
6

where *K*_*VM*_ denotes the von Mises kernel function, *κ* is the kernel bandwidth controlling the smoothness of the kernel and *I*_*0*_ is the Bessel function of the first kind of order 0. Here, we set *κ* = 8.21 which is equivalent to *σ* = *π*/9 in the normal distribution. The distances *d*_*ij*_ were discretized into 0.5 Å bins which span from 2 to 15 Å. The kernel density estimator is computed at π/9 grid points that are ranged from -π to π (in case of *ϕ*, from -π/2 to π/2).

The relative orientation between atoms is significantly affected by chain connectivity constrains when the atoms are positioned in residues that are close in the sequence. In order to reduce the chain (or bond) connectivity effect on the estimates of orientation-dependent probability, we applied a sequence separation as done in other studies [33, 40]. In this study, only atom pairs that are separated by at least 6 residues along the protein chain are considered.

Despite the use of kernel density estimation, in the case of rarely observed rotameric states in protein structures, there is still a problem of insufficient sample data. For example, the number of Ile rotamers in (+60°, +60°) dihedral pair is less than 1,000 in our database. In such case, rather than using poorly estimated probability density *p*^*obs*^(*θ*_*i*_|*d*_*ij*_, *R*_*i*_), we calculated the corrected probability density  as a linear combination of *p*^*obs*^(*θ*_*i*_|*d*_*ij*_, *R*_*i*_) and *p*^*obs*^(*θ*_*i*_|*d*_*ij*_):
7

where *N*(*d*_*ij*_, *R*_*i*_) is the number of observations used to estimate *P*^*obs*^(*θ*_*i*_|*d*_*ij*_, *R*_*i*_) and σ is a parameter that controls how many observations must be sampled such that both *P*^*obs*^(*θ*_*i*_|*d*_*ij*_, *R*_*i*_) and *P*^*obs*^(*θ*_*i*_|*d*_*ij*_) would have equal weights. Here we set σ = 1/100.

The expected probability distribution of angles can be calculated from a reference state in which the relative orientation of atom pair is determined randomly. Thus the expected probability is calculated by:
8

where *M* is a normalization factor such that the integration of *P*^*exp*^(*ϕ*) from -π/2 to π/2 becomes one. *P*^*exp*^(*ϕ*) is calculated numerically because there is no analytical way for integrating above equation.

### Interaction cutoff for ROTAS

**Figure 4:**
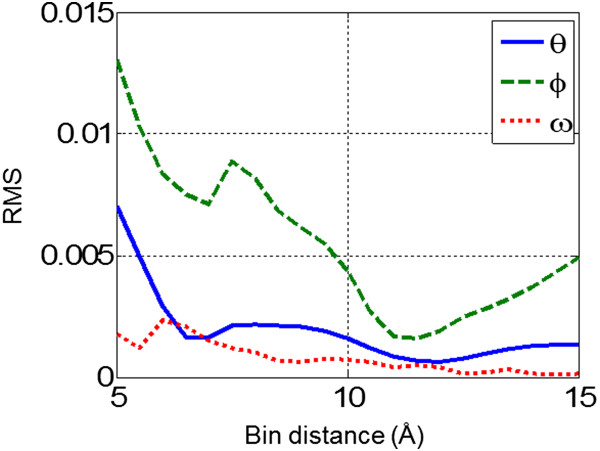
**The distance dependence of root mean square of (** ***P*** ^***obs***^ − ***P*** ^***exp***^ **) for angular parameters.** The observed probability distribution is calculated over all pairs of atom types. The thin, dashed and dotted curves corresponds to θ, *ϕ* and ω, respectively.

Although the distance bin between 14.5 and 15 Å was used as the cutoff in the construction of distance-dependent pairwise potential, we calculate the energy score within 10 Å and ignore the long-range tail of potentials beyond 10 Å. In fact, most physical interactions between atoms rapidly converge to zero beyond 8 ~ 10 Å. However, statistically derived potentials are likely to have fluctuations in the long-range, which inherently resulted from the statistical uncertainties. For example, Figures 4 reveals that the deviations of the observed probability from the expected probability for angular parameters do not consistently decrease as the atom-pair distance increases. It is noted that the root mean square of (*P*^*obs*^(*ϕ*|*d*) − *P*^*exp*^(*ϕ*|*d*)) increase after 12 Å. In addition, it was reported that distance-dependent pairwise potentials between hydrophobic atom pairs have either repulsive or attractive tail in the long range, even if no electrostatic interaction exists [7]. Thus it’s not always beneficial to include the long-range interactions in statistical potentials. We set the interaction cutoff to 10 Å without fine-tuning against a specific training dataset.

### Preparation of protein structures

We obtained a set of protein X-ray structures with a maximum R-factor of 0.25 and a resolution better than 2 Å from the protein sequence culling server, PISCES [54]. Also, protein chains were filtered out with a 40% sequence identity cutoff in order to have a set of non-homologous protein structures. A total 9321 protein structures were selected and downloaded from the Protein Data Bank (PDB) [55]. We did not attempt to exclude the homologous proteins to the test decoy sets from the 9321 proteins used for constructing the potential. It was reported that the exclusion has very little effect on the performance of statistical potentials [50]. The program REDUCE [56] was used to optimize the flip states of Asn, Gln, and His in all protein structures. Residues with multiple side-chain conformations were modified such that only the side-chain conformations with atoms having the highest occupancy and/or lowest temperature factors were used.

### Performance evaluation using decoy sets

We tested the ROTAS potential on various sets of decoys generated by different methods. A total of 13 decoy sets, including 4 state_reduced [57], fisa [58], fisa_casp3 [58], lmds [59], hg_structal, ig_structal, ig_structal_hires, lattice_ssfit [60], moulder [61], Rosetta [62], I-TASSER [39], AMBER99 [63] and CASP5-8 [64], were used The first 8 decoy sets were downloaded from the Decoys ‘R’ Us database [65] (http://dd.compbio.washington.edu/). The moulder decoy set produced by iterative target-template alignment and comparative-modeling methods was download from the Sali lab (http://salilab.org/decoys/). Three ab-initio simulation based decoy sets, Rosetta, I-TASSWER, Amber99 were obtained from http://zhanglab.ccmb.med.umich.edu/decoys/, and http://cssb.biology.gatech.edu/amberff99/, respectively. The CASP5-8 decoy set collected from the CASP5-CASP8 experiments was downloaded from http://zhanglab.ccmb.med.umich.edu/RW/ (cleaned version). The decoy models in this set were generated by a large variety of groups and methods participated in the CASP experiments.

The performance of ROTAS potential was compared to those of four other existing atomic potentials which take into account the orientation-dependencies on the interactions between atoms, blocks or side-chains: dDFIRE [38], OPUS_PSP [37], RWplus [39], and GOAP [40]. Furthermore, we compared ROTAS to evolutionary pairwise distance-dependent potential, EPAD [66] and attempted to combine both potentials to maximize the performance. The binary programs for these potentials were downloaded from the corresponding authors’ websites. Because ROTAS can be seen as an extended version of GOAP, we constructed our own GOAP potential energy function using the same structure database and techniques that were used for the construction of ROTAS. In this manner we reduced the possibility that estimation of probability distribution, specific computational implementation, or other technical aspects could affect the results, so that the improvements of ROTAS compared to GOAP can be fairly demonstrated.

The performance of statistical potentials is evaluated by four aspects: (1) the recognition of native structure from decoys, (2) the selection of the best (most native-like) decoy model, (3) the correlation between the energy score and model quality, and (4) the classification of near-native and non-native model. The quality of decoy models was assessed by TM-score which measures the similarity between two protein structures by a score between (0, 1] [67].

## Results and discussion

### The influence of rotameric states on atomic interactions

**Figure 5:**
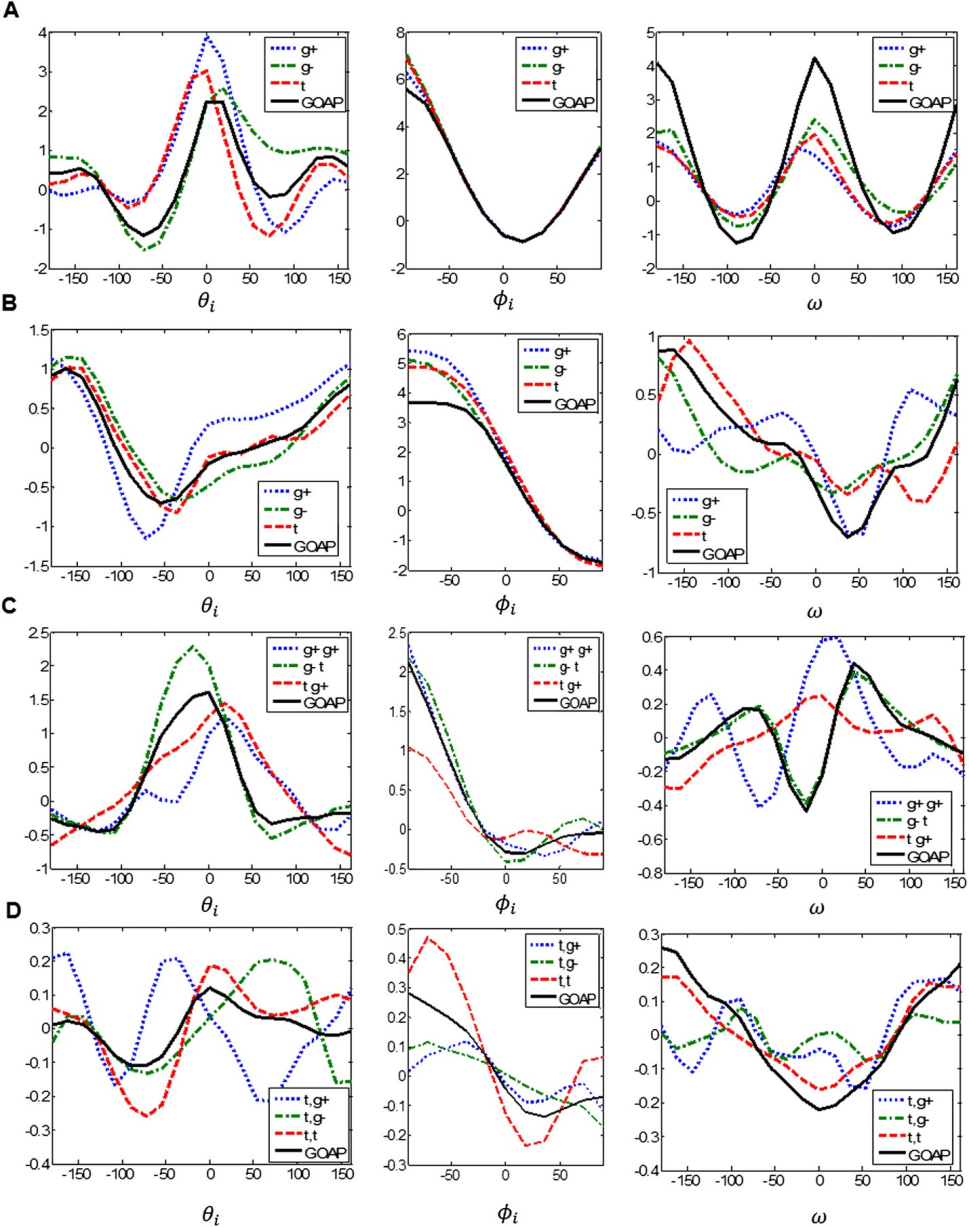
**Examples of the rotamer dependence of the energy terms in the ROTAS potential. (A)** Disulfide bond interaction for *i* and *j* = Cys S^γ^ at *d*_*ij*_ = 2 *Å*, **(B)** hydrogen bond interaction for *i* = Ser O and *j* = Gly N at *d*_*ij*_ = 3 *Å*, **(C)** nonpolar interaction for *i* = Ile C^γ2^ and *j* = Val C^γ1^ at *d*_*ij*_ = 5 *Å*, and **(D)** polar interaction for *i* = Lys N^ξ^ and *j* = Asp O^δ2^ at *d*_*ij*_ = 7 *Å*.

We constructed both ROTAS and GOAP potentials using the same structure database and techniques as described above. Figure 5 shows the energy profiles of ROTAS and GOAP for four different atom pairs. First of all, all examples clearly show that the energy profiles of ROTAS significantly vary depending on the rotameric state. While GOAP only reflects in some average sense the preferred orientation between interacting atoms, ROTAS adjusts the preferred orientation accurately depending on the rotameric state. The first example shows the disulfide interaction between Cys S^γ^ atoms (see Figure 5A). The torsional angular term  has two distinct favored positions regardless of the rotameric state. However, *E*(*θ*_*i*_|*d*_*ij*_, *R*_*i*_) shows slightly different curves. The most favored positions for θ_*i*_ are 90°, −72° and 72° for three rotameric states of Cys, g+, g-, and t, respectively. This might be due to close steric interactions between the backbone atoms and Cys S^γ^. The second example is a typical hydrogen bond interaction between Ser O and Gly N at a distance of 3 Å (see Figure 5B). It is observed that different relative position of Ser O^γ^ atom significantly affects on the hydrogen bond interaction between backbone atoms. Figure 5C shows an example of a non-polar interaction between Ile C^γ2^ and Val C^γ1^ at a distance of 5 Å. In this example, the GOAP potential shows very similar energy profiles with a particular rotameric state, (X1 = g- and X2 = t), which is the most populated rotamer for Ile (59% of Ile residues observed in this rotamer). The last example shows a polar interaction between Lys N^ξ^ and Asp O^δ2^ at a distance of 7 Å. It is noted that, although the pair distance is relatively longer rather than previous examples, the energy profiles of different rotameric states significantly differ. This suggests that the rotamer-dependency is not limited to short range interactions resulting from strong steric effects.

### Native structure recognition

We assessed the performance of ROTAS in terms of its ability for recognizing the native structures from decoy models and compared it with those of four other atomic statistical potentials. In this test, the performance was assessed by two measures: the number of targets having the native structure ranked as the lowest energy score and Z-score of the native structure. The Z-score represents the energy gap between the energy of native structure (*E*_*native*_) and the averaged energy of all decoys (〈*E*_*decoy*_〉) in units of the energy standard deviation of all decoys (*σ*_*decoy*_), which is defined as:
9

The lower the Z-score, the better the potential is for recognizing the native structures.

The results of the native structure recognition are summarized in Table 2. ROTAS could recognize total 409 native structures correctly out of 469 targets, which is the best success rate (87.2%) in the comparison. Although RWplus and GOAP record the highest success rate on I-TASSER and Amber 99, respectively, for the remaining 11 decoy sets, ROTAS recognized native structures more or equal than other potentials. GOAP recognized 399 native structures (85.1% success rate) with the average Z-score of −3.35. These results are consistent with those in the GOAP article which reported that the success rate and the average Z-score of GOAP are 81.3% (226 out of 278) and −3.57, respectively.

**Table 2:** **Performance on native structure recognition**

Decoy set	Targets	dDFIRE	OPUS_PSP	RWplus	GOAP	ROTAS
4state_reduced	7	7 (−4.15)	7 (−4.49)	6 (−3.50)	7 (−4.67)	7 (−**5.07**)
fisa	4	3 (−3.80)	3 (−4.24)	3 (−4.78)	3 (−3.98)	3 (−**4.83**)
lmds	10	6 (−2.44)	8 (−**5.63**)	7 (−1.03)	8 (−4.34)	8 (−5.47)
fisa_casp3	5	4 (−4.73)	5 (−6.33)	4 (−5.17)	4 (−6.65)	4 (−**7.48**)
hg_structal	29	15 (−1.25)	18 (−2.28)	12 (−1.70)	20 (−2.46)	**22** (−**2.51**)
ig_structal	61	26 (−0.82)	22 (−1.13)	0 (1.11)	44 (−1.91)	**46** (−**2.25**)
ig_structal_hires	20	16 (−2.00)	15 (−1.79)	0 (0.31)	18 (−2.68)	18 (−**3.11**)
lattice_ssfit	8	8 (−**10.08**)	8 (−6.56)	8 (−8.77)	8 (−7.94)	8 (−8.90)
moulder	20	18 (−2.74)	19 (−**4.83**)	19 (−2.84)	19 (−3.53)	19 (−3.76)
rosetta	59	12 (−0.43)	40 (−3.62)	20 (−1.21)	43 (−3.66)	**48** (−**4.18**)
I-TASSER	56	48 (−5.03)	49 (−5.40)	**56** (−5.77)	48 (−5.81)	49 (−**7.31**)
Amber99	47	27 (−3.42)	20 (−2.58)	16 (−2.38)	**38** (−4.38)	37 (−**4.48**)
CASP5-8	143	98 (−1.34)	134 (−**2.45**)	106 (−1.67)	139 (−2.26)	**140** (−2.43)
Total	469	288 (−2.16)	348 (−3.08)	257 (−1.98)	399 (−3.35)	**409** (−**3.80**)

Numbers outside the parentheses are the numbers of correctly recognized native structures and the ones in the parentheses are the average Z-scores of the native structures. The best scores are highlighted in bold type.

**Figure 6:**
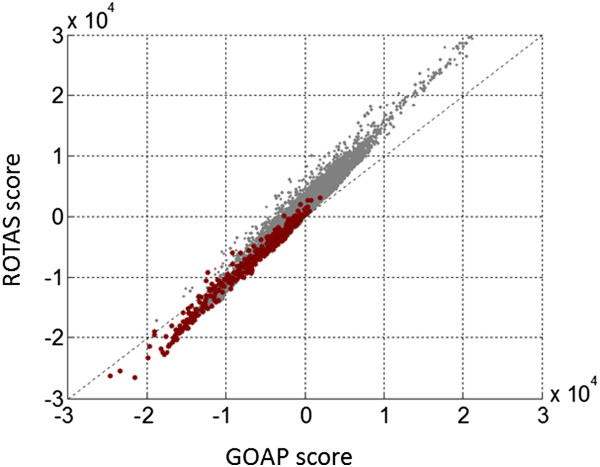
**Relationship between the energy scores of ROTAS and GOAP for all native and decoy structures.** Red and gray dots represent native and decoy structures, respectively.

The relative improvement of ROTAS over GOAP can be clearly seen in the average Z-scores. While GOAP correctly recognized the native structures comparable to ROTAS, it is noticed that ROTAS shows consistently improved Z-scores over all decoy sets tested here. Figure 6 shows the relationship between the energy scores of ROTAS and GOAP for all native (red) and decoy (gray) structures used in the test. It can be easily confirmed that ROTAS scores native structures with lower energies and decoy models with higher energies, compared to GOAP. We further investigated how this relationship can be affected by the interaction cutoff or the database used for deriving statistical potentials (see Additional file 1). We found that, as the interaction cutoff increases (e.g. > 10 Å), the correlation between ROTAS and GOAP scores decreases. However, the tendency that ROTAS gives lower scores to native structures than GOAP could be observed over different cutoffs. The use of different databases did not make a significant change in the relationship.

We found that the performance of ROTAS in native structure recognition is largely affected by experimental methods used to determine the native structures. The success rate of ROTAS is 89% for targets whose native structures were determined by X-ray crystallography, whereas the success rate significantly decreases to 60% when the native structures were determined by NMR spectroscopy (see Table 3). Furthermore, both the average success rate and Z-score decrease for low-resolution native structures. This might be because the ROTAS potential was constructed based on high-resolution X-ray structures. The large margin of error in the location of atoms in low-resolution structures (*e.g.*, > 2.2 Å) would decrease the confidence of computed energy score. This trend is also observed for other potential energy functions except RWplus which performs very well on NMR native structures. In fact, the RWplus potential can correctly recognize all 18 native NMR structures in the I-TASSER decoy set with low Z-scores.

**Table 3:** **The ability of ROTAS on native structure recognition as a function of native structure resolution**

Exp. method	Resolution	Targets	Rank1	Z
NMR	-	25	15 (60%)	−3.32
X-ray	all	444	394 (89%)	−3.82
	R < = 1.8	152	143 (94%)	−4.91
	1.8 < = R < 2.2	171	153 (89%)	−3.71
	2.2 < = R < 2.8	102	86 (84%)	−2.78
	2.8 < R	19	12 (63%)	−1.79

Numbers in parentheses are the ratio of Rank1 structures.

### Best model selection

We also assessed the ability of ROTAS in selecting the best models without native structures. This is more difficult and realistic task than the native structure recognition because, in practice, potential energy functions are used to find more and more native-like conformations in an iterative way when the native structure is not known. Thus, good potential energy function should be able to score the most native-like decoy model in the lowest energy. In this study, we use TM-score [67] to assess the quality of decoy models quantitatively. The TM-score measures the similarity between two protein structures by a score between (0, 1]. It is reported that TM-score is more accurate than other measures such as RMSD or GDT_TS because TM-score is sensitive to overall topology rather than local substructures [68].

Table 4 summarizes the result of the best model selection by dDFIRE, OPUS_PSP, RWplus, GOAP and ROTAS for 13 decoy sets. Measures log *P*_*B*1_ and log *P*_*B*10_ are the log probability of selecting the best (highest TM-score) model as the lowest energy model or among the top 10 lowest energy models, respectively. Suppose the top *i*^th^ scoring conformation *x*_*i*_ has the TM-score rank of *R*_*i*_ in *n* decoy models, then the log probability can be calculated as
10

**Table 4:** **Performance on best model selection**

Decoy set	dDFIRE		OPUS_PSP		RWplus		GOAP		ROTAS	
	logP _B1_	logP _B10_	logP _B1_	logP _B10_	logP _B1_	logP _B10_	logP _B1_	logP _B10_	logP _B1_	logP _B10_
4state_reduced	−3.60	−5.84	−4.03	**−6.14**	−2.80	−5.70	−4.68	−6.04	**−5.00**	−6.10
fisa	−2.68	−4.03	−1.57	−3.61	−2.18	−4.06	**−3.11**	−4.34	−2.23	**−5.19**
lmds	−1.51	−3.39	−1.08	−3.36	−1.04	−3.45	**−1.92**	**−3.57**	−1.83	−3.57
fisa_casp3	−1.42	−3.24	−0.81	−3.13	−1.19	**−4.23**	**−1.56**	−3.33	−1.30	−3.78
hg_structal	−2.44	**−3.33**	−2.55	−3.17	−2.50	−3.33	−2.42	−3.29	**−2.55**	−3.31
ig_structal	−2.06	−3.58	**−2.60**	**−3.76**	−2.14	−3.56	−2.17	−3.69	−1.96	−3.67
ig_structal_hires	−1.84	−2.66	−1.93	**−2.82**	**−1.95**	−2.81	−1.91	−2.71	−1.83	−2.77
moulder	−3.17	−4.79	−2.71	−4.62	−3.06	−4.90	**−3.84**	−5.08	−3.72	**−5.12**
lattice_ssfit	−1.60	−3.68	−1.03	−3.53	−1.13	**−4.10**	−1.24	−2.72	**−1.65**	−3.01
rosetta	−1.30	−3.45	**−1.76**	−3.18	−1.72	**−3.66**	−1.65	−3.56	−1.51	−3.59
I-TASSER	−1.83	**−3.87**	−1.26	−3.60	−1.78	−3.73	−1.77	−3.61	**−1.86**	−3.69
Amber99	−3.64	−5.43	−3.03	−4.72	−3.48	−4.94	−4.09	−5.64	**−4.25**	**−5.89**
CASP5-8	−1.89	−2.80	−1.36	−2.77	−1.88	**−2.81**	**−1.91**	−2.80	−1.87	−2.80
Total	−2.11	−3.58	−1.90	−3.44	−2.11	−3.56	**−2.26**	−3.60	−2.23	**−3.66**

The best scores are highlighted in bold type.

In both measures, GOAP and ROTAS shows better performance than other three potentials, dDFIRE, OPUS_PSP and RWplus. The average log *P*_*B*1_ by GOAP is slightly better than that by ROTAS, whereas the average log *P*_*B*10_ by ROTAS is better than that by GOAP. This indicates that the lowest energy model by GOAP is likely to be better in TM-score than that by ROTAS. However, when we consider the top 10 lowest energy models, ROTAS tend to include better TM-score decoy models in the top 10 than GOAP.

### Correlation between the energy score and decoy model quality

**Figure 7:**
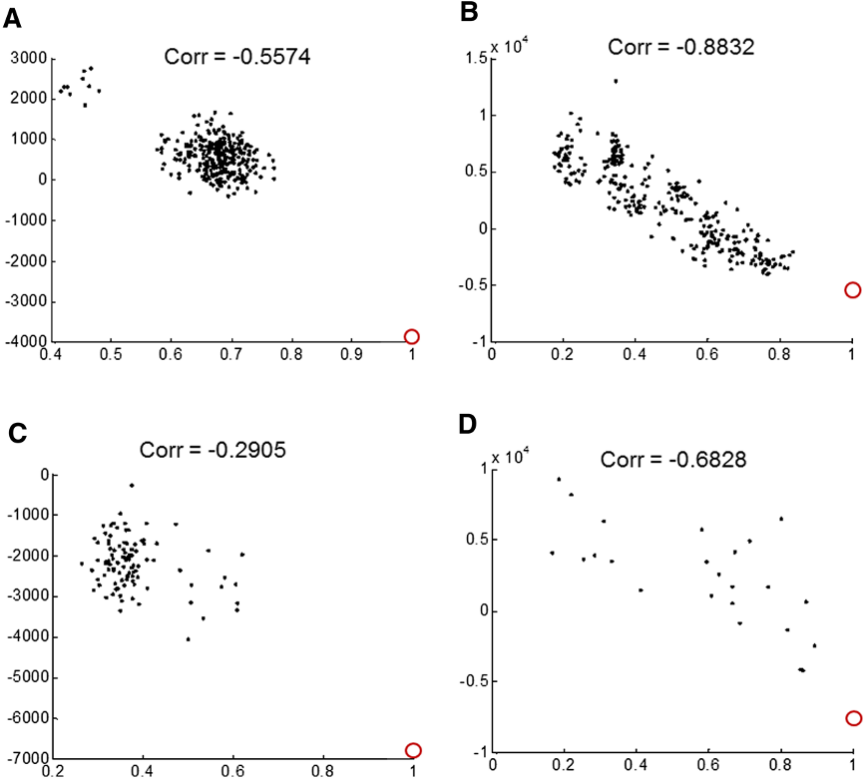
**Examples of Pearson correlation between ROTAS energy and TM-score. (A)** 1SCP_ in I-TASSER, **(B)** 1CAU in Moulder, **(C)** 1LOU in Rosetta and **(D)** T0324 in CASP7. The native structures are included and represented as empty red circle at TM-score = 1.

Next, we examined the correlation of the energy score and the quality of decoy models in order to assess the ability of ROTAS in guiding conformation sampling to near-native states. In an energy landscape perspective, a good potential energy function should not only be able to make a deep energy minimum with steep wall at the native state but also be able to form a middle-range funnel biased toward the native state. In Table 5, we compare the performance of potentials as assessed by both their Pearson correlation coefficient (*r*) and the Kendall’s rank correlation coefficient (τ) between the energy score and TM-score. Overall, the performance of potentials does not show significant difference depending on the correlation measures. We find that ROTAS shows the best performance in both measures. GOAP yields the second best performance in the average correlation coefficients. dDFIRE and RWplus have comparable performance although the average correlation coefficients of RWplus is slightly better than those of dDFIRE. OPUS_PSP performs significantly worse than the other potentials tested although its performance comes in third in the native structure recognition. Figure 7 shows some examples of the correlation between ROTAS energy and TM-score from different decoy sets.

**Table 5:** **Performance on correlation coefficients between energy score and model quality**

Decoy set	dDFIRE		OPUS_PSP		RWplus		GOAP		ROTAS	
	r	τ	r	τ	r	τ	r	τ	r	τ
4state_reduced	−0.693	−0.483	−0.590	−0.399	−0.605	−0.417	−0.766	−0.550	**−0.783**	**−0.562**
fisa	−0.461	−0.321	−0.282	−0.189	−0.462	−0.315	**−0.476**	**−0.327**	−0.442	−0.297
lmds	**−0.248**	**−0.168**	−0.091	−0.054	−0.147	−0.095	−0.228	−0.149	−0.227	−0.149
fisa_casp3	**−0.251**	**−0.168**	−0.090	−0.063	−0.236	−0.152	−0.161	−0.102	−0.182	−0.117
hg_structal	−0.796	−0.618	−0.752	−0.553	−0.806	**−0.630**	−0.808	−0.609	**−0.811**	−0.602
ig_structal	−0.766	−0.308	−0.779	−0.340	−0.782	−0.277	**−0.851**	**−0.377**	−0.836	−0.372
ig_structal_hires	−0.844	−0.373	−0.832	−0.403	−0.879	−0.411	**−0.890**	**−0.436**	−0.860	−0.401
lattice_ssfit	−0.068	−0.047	−0.050	−0.033	**−0.096**	**−0.059**	−0.034	−0.025	−0.043	−0.029
moulder	−0.832	**−0.670**	−0.755	−0.600	−0.792	−0.642	−0.823	−0.660	**−0.833**	−0.665
rosetta	−0.265	−0.176	−0.192	−0.113	−0.350	**−0.237**	−0.330	−0.212	**−0.351**	−0.221
I-TASSER	**−0.522**	**−0.303**	−0.281	−0.195	−0.485	−0.290	−0.465	−0.276	−0.456	−0.271
Amber99	−0.609	−0.339	−0.421	−0.201	−0.526	−0.313	−0.692	−0.355	**−0.721**	**−0.357**
CASP5-8	−0.594	−0.488	−0.440	−0.354	−0.611	−0.501	−0.593	−0.490	**−0.613**	**−0.502**
Total	−0.581	−0.380	−0.465	−0.297	−0.584	−0.382	−0.603	−0.394	**−0.612**	**−0.396**

r: Pearson’s correlation coefficient.

τ: Kendall’s rank correlation coefficient.

The best scores are highlighted in bold type.

### Classification of near-native and non-native model

In order to compare the performance of ROTAS and other potentials in a more robust way, we evaluated the performance of statistical potentials using receiver operating characteristic (ROC) technique [69]. That is, the energy score was used to rank the decoy models for each target, and then thresholds were applied to classify a group of near-native models among a pool of putative models. The near-native (positive) were defined as those with TM-score larger than 0.5 with respect to the native structure, and non-native (negative) models otherwise. In fact, it is reported that protein structures having a TM-score > 0.5 are mostly in the same fold [68]. ROC curves were obtained by plotting the true positive ratio against the corresponding false positive ratio for all thresholds on the energy score.

We computed the area under the ROC curve (AUC) which provides a robust measure of accuracy over the whole range of thresholds. In the context of this test, the AUC represents the probability that a potential energy function scores a randomly chosen near-native (positive) model lower than a randomly chosen non-native (negative) model. Table 6 presents the results of the classification test. The average AUC for each decoy set is shown. We performed this classification test only on targets having a sufficient number of near-native models (>10). The four decoy sets including hg_structal, ig_structal, ig_structal_hires, and lattice_ssfit were excluded, accordingly. Although RWplus and dDFIRE give the best average AUCs for one or two decoy sets, ROTAS provides the best classification performance against all other decoy sets. Thus, the highest average AUC for all targets is obtained by ROTAS.

**Table 6:** **The area under the ROC curves for classification of near-native and non-native model**

	Targets	<|P|>	<|N|>	dDFIRE	OPUS_PSP	RWplus	GOAP	ROTAS
4state_reduced	7	195	468	0.86	0.80	0.81	0.91	**0.92**
fisa	2	47	453	0.79	0.60	**0.79**	0.79	0.77
lmds	2	60	439	**0.74**	0.64	0.66	0.61	0.56
fisa_casp3	2	20	1672	**0.74**	0.58	0.72	0.68	0.70
moulder	19	151	169	0.95	0.93	0.95	0.95	**0.96**
rosetta	27	50	50	0.71	0.66	0.74	0.75	**0.77**
I-TASSER	31	229	217	0.79	0.71	0.77	0.80	**0.80**
Amber99	41	219	821	0.87	0.79	0.83	0.93	**0.93**
CASP5-8	89	14	7	0.82	0.75	0.84	0.83	**0.84**
Total	220	105	245	0.82	0.75	0.82	0.84	**0.85**
p-value				1.02E-04	7.70E-27	1.30E-03	2.03E-06	

<|P|>: Averaged number of positive (near-native) models in each target.

<|N|>: Averaged number of negative (non-native) models in each target.

p-value: P value of paired t-test of the difference of the AUC between ROTAS and the given potential.

The best scores are highlighted in bold type.

To quantify the statistical significance of the difference between ROTAS and other potentials, P values of the paired t-test of the differences between ROTAS and other potentials for the AUCs were also calculated. Cleary, ROTAS gives statistically significant (P value < 0.01) better results than all other potentials.

So far, the results showed that ROTAS performs better than other competing potentials not only in native structure recognition, but also in best model selection and correlation coefficients between energy and model quality. The following sections discuss factors affecting on the performance of ROTAS as well as a possible way to improve the performance by combining other statistical potentials.

### Interaction cutoff effect on the performance

**Figure 8:**
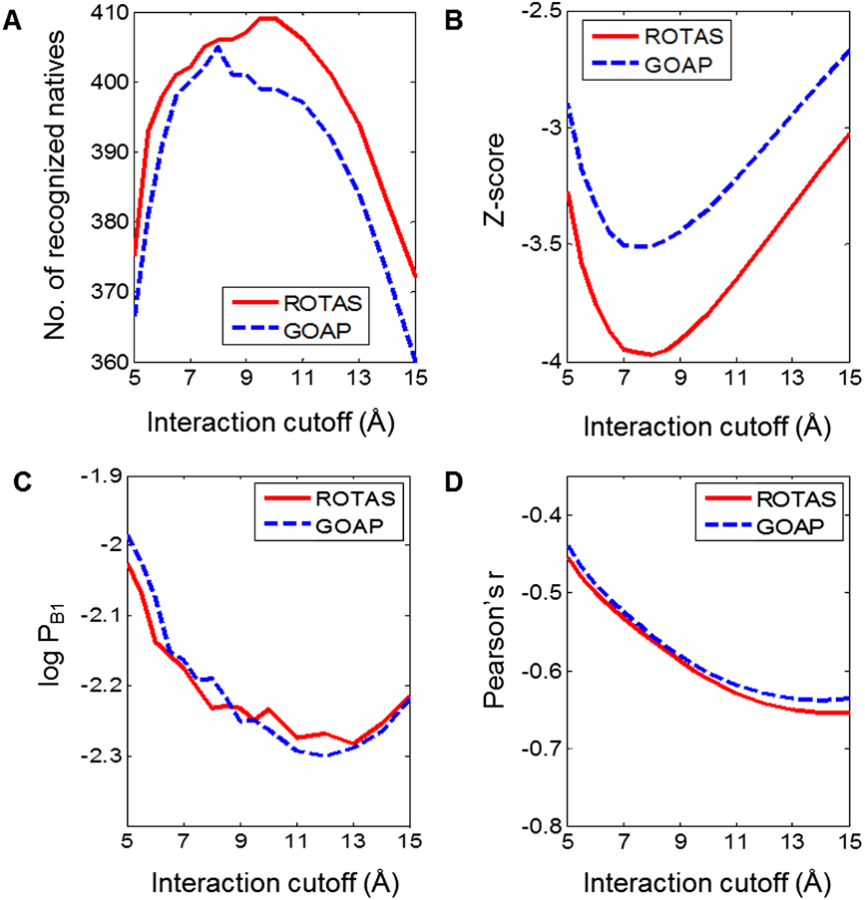
**Influence of the cutoff distance on the performance of ROTAS and GOAP. (A)** Number of first-ranked native structures, **(B)** Average Z-score of the native structures, **(C)** Average log *P*_*B*1_ and **(D)** Average Pearson’s correlation coefficient between TM-score and energy score.

The interaction cutoff effect on the performance of ROTAS and GOAP was examined. The performances of ROTAS and GOAP are significantly affected by the interaction cutoff (see Figure 8). Interaction cutoffs between 7 and 10 Å maximize the number of correctly recognized native structures and minimize the average Z-score for both potentials. Increasing or decreasing the cutoff outside of this range makes the performance for native structure recognition worse dramatically. The performance of ROTAS and GOAP for recognizing the best models is maximized around 11 ~ 13 Å. On the other hand, as the interaction cutoff increases, the average correlation coefficient decreases. But the slopes around 13 ~ 15 Å are almost zero. Although the optimal interaction cutoff varies depending on the evaluation criteria, we confirm that the long-range interactions in statistical potentials could reduce the performance of potentials and an interaction cutoff of 10 Å for ROTAS gives a moderate performance on various evaluation criteria. It should be noticed that even though optimal interaction cutoffs are applied to individual potentials, ROTAS performs better than GOAP.

It is noticed that the highest average correlation coefficient is obtained when we consider all the long-range interactions available in the potentials. However, in this case, the native structures are poorly recognized. A similar observation that a scoring function producing a good linear correlation is normally less capable of recognizing the native state has been reported in a previous study [70]. A theoretical study argue that the potential energy of near-native conformations might not be linearly related to their distances from the native state [71]. Also, since a shorter interaction cutoff would increase ruggedness of the energy landscape [72], the energy score of decoy models might be affected by small structural differences sensitively.

### Different reference states

We applied five widely-used reference states including DFIRE, DOPE, RW, RAPDF and KBP for the distance-dependent pairwise potential in ROTAS and compared their performances. To rigorously compare the influence of the reference state on the performance, we constructed all five distance-dependent pairwise potentials using the same structure database, the same cutoff distance, and the same bin width. Table 7 summarizes the performance results on the 13 decoy sets. It is not clear to find the best reference state outperforming other reference states. In terms of Rank1, there is little difference on the performance. Each reference state shows strength on difference evaluation criteria as incorporated into ROTAS. The RAPDF reference state gives the best average Z-score whereas the DFIRE reference state shows the best average log *P*_*B*10_. The RW reference state shows the best performance on log *P*_*B*1_ and both correlation measures. Overall, the DFIRE and RW reference states are found to show better performance than other three reference states in ROTAS.

**Table 7:** **Comparison of different reference states in ROTAS**

Ref. state	Rank1	Z-score	logP _B1_	logP _B10_	Pearson’s r	Kendall’s τ
DFIRE	409	−3.795	−2.233	**−3.656**	−0.612	−0.396
DOPE	409	−3.810	−2.172	−3.576	−0.566	−0.358
RW	408	−3.818	**−2.258**	−3.645	**−0.617**	**−0.401**
RAPDF	409	**−3.867**	−2.185	−3.592	−0.578	−0.367
KBP	409	−3.638	−2.276	−3.630	−0.609	−0.393

The best scores are highlighted in bold type.

### Possible improvement by incorporating evolutionary information

Beyond structural features embedded in known protein structures, evolutionary information also can be utilized in protein structure prediction [73]. Evolutionary pairwise distance-dependent potential (EPAD) [66] is a successful example of statistical potentials utilizing evolutionary information in a large amount of sequence data. In fact, EPAD has different energy profile between two atoms depending on the protein under consideration and the sequence profile context of the atoms (i.e. evolutionary information). As a possible way to improve ROTAS, we built a composite energy function by replacing the distance-dependent pairwise energy term in ROTAS with EPAD.

Table 8 compares the performance of EPAD, ROTAS and the composite energy function, EPAD + ROTAS. It was confirmed that ROTAS could improve the performance in native structure recognition when incorporating EPAD. It correctly recognized 417 native structures, 7 more than ROTAS alone. The average Z-score was also improved. However, in correlation coefficients, EPAD shows the best performance, which indicates that EPAD would be good for ab initio folding. It should be noted that, in EPAD + ROTAS, we did not fine-tune weights for energy terms (i.e. equal weight). In fact, EPAD ignores side-chain atoms in energy calculation (i.e. backbone-based potential), while ROTAS takes all atoms into account. Thus, it would be desirable to adjust weights for ROTAS and EPAD to maximize the performance when building a composite energy function.

**Table 8:** **Performance of EPAD, ROTAS and ROTAS + EPAD**

	Rank1	Z-score	logP _B1_	logP _B10_	Pearson’s r	Kendall’s τ
EPAD	260	−2.13	−2.11	−3.56	**−0.68**	**−0.45**
ROTAS	409	−3.80	**−2.23**	**−3.66**	−0.61	−0.40
EPAD + ROTAS	**416**	**−4.17**	−2.22	−3.61	−0.59	−0.38

The best scores are highlighted in bold type.

## Conclusions

In this study, we hypothesized that the rotameric state of residues critically affects on the specificity of non-bonded interactions within protein structures. This idea was applied to develop a new multibody statistical potential (ROTAS) for protein structure prediction. The interaction between two atoms is specified by not only the distance and relative orientation but also by two state parameters concerning the rotameric state of the residues to which the interacting atoms belong. It was clearly found that the rotameric state is correlated to the specificity of atomic interactions. Furthermore, such rotamer-dependencies are not limited to specific type or certain range of interactions.

The incorporation of accurate modeling of residue flexibility has been shown to be a possible means of improving the specificity of potential energy functions. We tested ROTAS using various decoy sets and compared its performance to those of several existing atomic statistical potentials which incorporate orientation-dependent energy terms. For a fair comparison, we implemented our own GOAP potential using the same structure database and techniques used for the construction of ROTAS. The results showed that ROTAS performs better than other competing potentials not only in native structure recognition, but also in best model selection and correlation coefficients between energy and model quality. In particular, the relative improvement of ROTAS over GOAP indicates that the rotameric state of residues can be incorporated for a fine-tuning of atomic-level statistical potentials. The effectiveness of ROTAS may provide insightful information for the development of many applications which require accurate side-chain modeling such as homology modeling, protein design, mutation analysis, protein-protein docking and flexible ligand docking.

## Electronic supplementary material

Additional file 1: **The effects of sample database and interaction cutoff on the relationship between ROTAS and GOAP scores.** Two different databases, each of which includes 6,000 protein structures randomly selected from our database, are used to derive GOAP and ROTAS. (DOCX 107 KB)
