# Supplementary material for: ROTAS: a rotamer-dependent, atomic statistical potential for assessment and prediction of protein structures
Source: BMC Bioinformatics. 2014 Sep 18;15(1):307. doi: 10.1186/1471-2105-15-307 (PMC4262145; doi:10.1186/1471-2105-15-307)
Supplement: Supplementary file 1 — Additional file 1: The effects of sample database and interaction cutoff on the relationship between ROTAS and GOAP scores. Two different databases, each of which includes 6,000 protein structures randomly selected from our database, are used to derive GOAP and ROTAS. (DOCX 107 KB) [file 12859_2014_6637_MOESM1_ESM.docx]

**The effects of sample database and interaction cutoff on the relationship between ROTAS and GOAP scores.** Two different databases, each of which includes 6,000 protein structures randomly selected from our database, are used to derive GOAP and ROTAS.

|  | Database 1 | Database 2 |
| --- | --- | --- |
| Interaction  cutoff = 8 |  |  |
| Interaction  cutoff = 12 |  |  |
| Interaction  cutoff = 15 |  |  |
